# Late initiation of antenatal care among pregnant women in Addis Ababa city, Ethiopia: a facility based cross-sectional study

**DOI:** 10.1186/s12905-022-02148-4

**Published:** 2023-01-10

**Authors:** Niguse Girma, Meyrema Abdo, Sultan Kalu, Afework Alemayehu, Teshale Mulatu, Tahir Ahmed Hassen, Kedir Teji Roba

**Affiliations:** 1 Department of Public Health, Adama Hospital Medical College, Adama, Ethiopia; 2 School of Nursing and Midwifery, College of Health and Medical science, Haramaya University, Harar, Ethiopia

**Keywords:** Prenatal care, Pregnancy, Pregnant women, Antenatal care, Timing, Ethiopia

## Abstract

**Background:**

Antenatal care (ANC) is the care given to pregnant women to prevent poor feto-maternal outcomes during pregnancy. The World Health Organization recommends first ANC visit be started as early as possible within in 12 weeks of gestation. Although there is improvement in overall ANC coverage, a sizable proportion of pregnant women in Ethiopia delay the time to initiate their first ANC visit. Therefore, this study aimed to investigate factors associated with late ANC initiation among pregnant women attending public health centers in Addis Ababa, Ethiopia.

**Methods:**

A facility-based cross-sectional study was conducted among 407 randomly selected pregnant women who attended ANC at selected public health centers in Addis Ababa from December 2020 to January 2021. Data were collected using pretested and structured questionnaires through a face-to-face interview and reviewing medical records. Binary and multivariable logistic regressions were fitted sequentially to identify predictors for late ANC initiation. Adjusted odds ratios with 95% CI were computed to measure the strength of associations and statistical significance was declared at a *p*-value < 0.05.

**Result:**

This study showed that 47% of pregnant women started their first ANC visit late.The age of 30 years and above, being married, unplanned pregnancy, having a wrong perception about the timing of the first ANC visit, and not having ANC for previous pregnancy was significantly associated with late ANC initiation.

**Conclusion:**

Nearly half of the women initiated their first ANC visit late. Tailored interventions aimed at promoting early ANC initiation should target married women, women with an unplanned pregnancy, women who perceived the wrong timing of their first ANC, and those who have no ANC for their previous pregnancy.

**Supplementary Information:**

The online version contains supplementary material available at 10.1186/s12905-022-02148-4.

## Background

Antenatal care (ANC) is care given by trained health-care providers to pregnant women for the well-being of the mother and the fetus during pregnancy. The World Health Organization (WHO) recommends that ANC should be initiated within 12 weeks of gestation and considers “late initiation” when it is commenced after 12 weeks of gestation [1].

In 2017, nearly 295 000 mothers died from pregnancy and childbirth-related complications, which could be translated to 810 maternal deaths every day. The vast majority of these deaths (94%) occurred in low-income countries, of which Sub-Saharan Africa contributed to approximately 66% of deaths [2, 3]. Ethiopia is one of the Sub-Saharan African countries that contribute to this unacceptable high number of maternal deaths in the region with a maternal mortality ratio (MMR) of 412/per 100,000 live births [4].

Between 2000 and 2017, the maternal mortality rate was reduced only by 38% worldwide. Although reducing maternal deaths has remained a global priority, the progress towards the global target of reducing maternal mortality ratio (MMR) to 70 per 100,000 livebirths or less by 2030 is very slow, indicating the need for early risk identifications and management of pregnancy-related complications during ANC follow up [2, 3].

In 2016, the WHO approved a new ANC model with increased numbers of contacts from four visits to eight contacts, intending to reduce prenatal mortality and improve pregnancy outcomes. Recent evidence shows that eight or more ANC contacts can decrease perinatal deaths by 8 per 1000 births in comparison to four visits [5, 6].

In addition to the number of ANC contacts, the timing of its initiation is also an important factor for feto-maternal outcomes. Timely initiation of ANC visits serves as a gateway for risk identification and early diagnosis of pregnancy-related complications to improve pregnancy outcomes [7, 8]. For example, studies showed that regular antenatal care from a skilled provider reduces maternal mortality by 20% [9]. On the contrary, delaying ANC visits or lack of ANC follow-up is associated with adverse feto-maternal outcomes such as premature birth, low birth weight, stillbirth, and increased risk of maternal complications during pregnancy, childbirth, and the puerperium [9–12].

Evidence indicated that globally 43% of pregnant women initiate their first ANC visit timely, although there is a huge discrepancy between developed and developing regions [13]. For example, compared to the developed region, where 85% of pregnant women start their ANC follow-up in the first trimester, only 45% of pregnant women start the follow-up in the first trimester in developing regions, and this stands at 25% for Sub-Saharan Africa [14].

In Ethiopia, according to the Ethiopian Demography and Health Survey report (EDHS, 2016), 62% of the women had at least one ANC visit and only 20% of women had their first antenatal care visit during the first trimester [4].

The Ethiopian government, in its Health Sector Transformation Plan (2015/16–2019/20), set a goal to achieve 95% ANC utilization of at least 4 visits and adopted the WHO-focused ANC model in combination with the eight contacts to alleviate the problems related with late ANC initiation [15]. Yet, despite the efforts made by the government to achieve high coverage of ANC visits, the proportion of pregnant women starting their first ANC follow-up within the first trimester has remained low.

It has been reported that factors such as lack of education, distance from a health facility, low income, unplanned pregnancy, and previous obstetrics history are associated with the late initiation of ANC [16–18]. Furthermore, the coverage of ANC follow-up or its early initiation might be associated with some unmeasured factors. Investigating factors associated with late ANC initiation comprehensively in the such setting would be important for a program design and interventions. This study, therefore, aimed to investigate factors associated with late ANC initiation among pregnant women attending public health centers in Addis Ababa, Ethiopia.

## Methods and materials

### Study area and period

The study was conducted in Addis Ababa, the capital city of Ethiopia. There are eleven sub-cities in Addis Ababa, with over five million total population as of 2021. According to Federal Ministry of Health, there are 10 general Hospitals, two specialized hospitals, 36 private hospitals, 700 clinics and 35 non-governmental organizations and about 96 government health centers offering services to the dwellers of the city and the surrounding community [19]. This study was conducted from December 2020 to January 2021.

### Study design and population

A facility-based cross-sectional study was conducted among pregnant women who came to attend their first ANC visit at selected public health centers during the study period. Pregnant women who came at below eight weeks of gestation and who were mentally ill were excluded from the study.

### Sample size determination and sampling technique

The sample size was estimated using a single population proportion formula, with the assumption of proportion (P = 59.8%) [20], 95% Confidence interval, and 5% margin of error.

$$n =\frac{{\left(Z\frac{{\upalpha }}{2}\right)}^{2}P(1-P)}{{d}^{\begin{array}{c}\\ 2\end{array}}}$$

*n* = (1.96)^2^ × 0.598 (1−0.598)/ (0.05)^2^.

*n* = 3.8416 × 0.596 × 0.404/0.0025, *n* = 369.9 ~ 370.

Considering 10% non-response rate, the final sample size was 407.

A simple random sampling technique was used to select eleven health centers, that is, one health center from each sub-city. For each selected health center, the average number of pregnant women was obtained from the ANC registration book. The total sample size was proportionally allocated to each health center based on the average number of pregnant women attending the ANC clinic. Then systematic random sampling was employed to select the study participants from each health center. During the study period, a total of 1016 pregnant women visited the selected health centers for ANC follow. The interval (Kth) for the systematic random sampling method was obtained dividing the number of pregnant women by the sample size which yielded three (1016/407 = 3). The first study participant was selected using lottery method and then every 3rd pregnant woman were enrolled to the study.

### Data collection procedure and instrument

The data were collected through face-to-face interview using structured questionnaire adapted from existing literatures [16, 21–36]. The questionnaire contains questions on socio-demographic characteristics, obstetric factors, health service-related factors, and knowledge and attitude towards ANC (Additional file 1). Two days of training on the data collection tools and procedures was given for data collectors and supervisors. The questionnaire was pretested on 5% of the sample size, out of the selected health centers, and necessary adjustments were made before using it for the actual data collection. The validity of the questionnaire was assessed using face validity/content validity and the inter-rater reliability test was performed to check the reliability. The data were collected by five diploma holder nurses and supervised by two bachelor of science nurses who were working out of the selected study facilities. Gestational age was estimated by asking the women their last normal menstrual period (LNMP), and further medical records of the women were reviewed to obtain some information including obstetric history, gestational age estimation either from ultrasound reports or manual fundal height measurement if the women were unable to recall their LNMP.

### Data processing and analysis

Data were checked for completeness and consistency. The cleaned data were coded and entered to Epi Info and analyzed using SPSS version 22. Descriptive statistics were computed to present the results of independent variables. The outcome variable was dichotomized as early versus late ANC initiation. A woman was considered to have early initiation of ANC if she commenced first ANC visit within the first trimester at or between eight and 12 weeks of pregnancy and considered to have a late ANC visit, otherwise [28].

A binary logistic regression models were used to assess the association between independent and outcome variables (late ANC initiation). The variables with P*-value* < 0.25 in a bivariate analysis were included in multivariable analysis to control for confounding effect. Hosmer and Lemeshow goodness‑of‑fit test was done to assess the fitness of the model during multivariable analysis. Adjusted odds ratios (AOR) with 95% CI were used to measure the strength of association between independent variables and outcome variable and level of significance was set at P value less than 0.05.

## Results

### Socio-demographic characteristics of the respondents

Out of 407 pregnant women, 401 included in this study, giving the response rate of 98.5%. The mean age of respondents was (28 ± 5 SD) years. Three hundred thirty-six (84%) of the respondents were married and 154 (38.4%) attended secondary school and above level.

A half of the respondents who delayed the timing of first ANC initiation were in the age group of 30 years and above (Table 1).

**Table 1:** Socio-demographic characteristics of the respondents (*n* = 401)

Variable	Timing of ANC initiation		Total (%)
	Early (%)	Late (%)	
Age (in years)			
15–19	13 (6.1)	13 (6.9)	26 (6.5)
20–24	60 (28.2)	34 (18.1)	94 (23.4)
25–29	72 (33.8)	46 (24.5)	118 (29.4)
30 and above	68 (31.9)	95 (50.5)	163 (40.6)
Ethnicity			
Amhara	76 (35.7)	41 (21.8)	117 (29.2)
Oromo	53 (24.9)	57 (30.3)	110 (27.4)
Gurage	31 (14.6)	37 (19.7)	68 (17.0)
Tigre	30 (14.1)	25 (13.3)	55 (13.7)
Others^a^	23 (10.8)	28 (14.9)	51 (12.7)
Religion			
Orthodox	121 (56.8)	86 (45.7)	207 (51.6)
Muslim	48 (22.5)	45 (23.9)	93 (23.2)
Protestant	43 (20.2)	47 (25.0)	90 (22.4)
Others^b^	1 (0.5)	10 (5.3)	11 (2.7)
Marital status			
Single	22 (10.4)	42 (22.3)	64 (16.0)
Married	190 (89.6)	147 (77.7)	337 (84.0)
Educational Status			
Illiterate	48 (22.5)	55 (29.3)	103 (25.7)
Primary education	64 (30.0)	80 (42.6)	144 (35.9)
Secondary and above	101 (47.4)	53 (28.2)	154 (38.4)
Occupation			
Self employed	94 (25.3)	106 (57)	200 (50.1)
Government employee	51(23.9)	27 (14.5)	78 (19.5)
House wife	60 (28.2)	47 (25.3)	107 (26.8)
Others^c^	10 (3.8)	6 (3.2)	16 (3.5)
Monthly income (Ethiopian Birr)			
500 and below	25 (12.4)	18 (9.9)	43 (11.2)
501–1000	38 (18.8)	53 (29.1)	91 (23.7)
1001–1500	28 (10.4)	34 (13.2)	62 (11.7)
Above 1500	118 (58.4)	87 (47.8)	205 (53.4)

^a^Wolaita, Sidama, and Hadiya;

^b^Catholic, Adventist, and Pagans;

^c^Daily laborer and housemaid.

### Obstetrics history of the respondents

Of the total respondents, 268 (66.8%) reported to have 1–2 parity. About one fifths of respondents reported to have experienced abortion and 20 (5%) reported to encounter child death (Table 2).

**Table 2:** Obstetrics history of respondents

Variable		Timing of ANC initiation				Total (%)	
		Late (%)		Early (%)			
Parity (*N* = 401)							
1 − 2		131 (69.7)		137 (64.3)		268 (66.8)	
3 − 4		52 (27.7)		69 (32.4)		121(30.2)	
5 and above		5 (2.7)		7 (3.3)		12 (3.0)	
History of abortion (*N* = 401)							
No		150 (79.8)		172 (80.8)		322 (80.3)	
Yes		38 (20.2)		41(19.2)		79 (19.7)	
Types of abortion (*N* = 79)							
Spontaneous		27 (84.4)		40 (85.0)		67 (84.8)	
Induced		5 (15.6)		7 (15.0)		12 (15.2)	
History of child death (*N* = 401)							
Yes		11(7.9)		9 (3.4)		20 (5.0)	
No		129 (92.1)		252 (96.6)		381(95.0)	
Gestational age (*N* = 401)							
≤ 12 weeks		71(35.5)		142 (71.0)		213 (53.1)	
> 12 weeks		129 (64.5)		59 (29.0)		188 (46.9)	

### History of current pregnancy and timing of first ANC visit

Of 401 respondents, 188 (46.9%) started their first ANC late. One hundred sixty-one (40.2%) respondents confirmed their current pregnancy when menstruation period was late for two months and 119 (29.8%) confirmed their pregnancy by urine test offered by a health care provider. Among respondents, 339 (85%) reported that their current pregnancy was planned and 60 (15%) reported that the pregnancy was unplanned (Table 3).

**Table 3:** History of current pregnancy of the respondents

Variable	Timing of ANC initiation		Total (%)
	Late (%)	Early (%)	
Methods of pregnancy confirmation			
Delayed menstrual period for one month	43 (23.0)	47 (22.0)	90 (22.6)
Delayed menstrual period for two months	71 (37.4)	91(42.8)	162(40.4)
Delayed menstrual period for three months	20 (10.7)	10 (4.7)	30 (7.4)
By pregnancy test	54 (28.9)	65 (30.5)	119 (29.7)
Planned pregnancy			
No	31 (16.6)	29 (13.6)	61 (15.2)
Yes	156 (83.3)	184 (86.4)	340 (84.8)
Received advice from someone on ANC			
No	33 (17.6)	31 (14.6)	64 (16.0)
Yes	154 (82.4)	183 (85.5)	337 (84.0)
Reason for deciding to start ANC follow up			
Perceived appropriate time	135 (71.7)	145(68.1)	280 (69.8)
From-previous experience	31 (16.6)	45 (21.1)	76 (19.0)
Pregnancy-danger signs	5 (2.7)	10 (4.7)	15 (3.8)
*Others	17 9.0)	13 (6.1)	30(7.5)
Transport cost paid for getting health service Yes No	25 (13.3) 163 (86.7)	45 (21.1) 168 (78.9)	70 (17.5) 331(82.5%)

*Others  Advice from relatives, partners and family

### Perception of the respondents on ANC services utilization

Three hundred fourteen (78.3%) of the respondents perceived that ANC is highly important to the health of mother and about 72% perceived that ANC is highly important for the fetus. Regarding the correct time of ANC booking, 242 (62.7%) of the women perceived that ANC should be initiated within or before 12 weeks of gestation and 144 (37.3%) perceived that ANC should begin after 12 weeks of gestation. Most of respondents, 249 (62%), had previous ANC follow up (Table 4).

**Table 4:** Perception of respondents towards about the benefits and timing of ANC initiation

Variable	Timing of ANC initiation		Total (%)
	Late (%)	Early (%)	
Importance of ANC for your own health			
Highly important	137 (72.9)	177 (83.1)	314 (78.3)
Medium	48 (25.5)	33 (15.5)	81 (20.2)
Less important	3 (1.6)	1 (0.5)	4 (1.0)
I don’t know	1(0.5)	1 (0.5)	2(1)
Importance of ANC for the fetus			
Highly important	128 (68.4)	161 (75.6)	289 (72.2)
Medium	58 (31.0)	45 (21.1)	103 (25.8)
Less important	1 (0.5)	5 (2.3)	6 (1.2)
I don’t know	1 (0.5)	2 (0.9)	3 (0.8)
Perceived timing of the first ANC visit			
≤ 12 weeks	69 (35.8)	173 (83.2)	242 (60.3)
> 12 weeks	124 (64.2)	35 (16.8)	159 (39.6)
Previous ANC follow up			
No	100 (54.1)	48 (22.6)	152 (38.0)
Yes	85 (45.9)	164 (77.4)	249 (62.0)

### Factors associated with late initiation of ANC

In a bivariate analysis, maternal age, marital status, educational status, occupation, transportation cost for ANC, pregnancy intention, perceived timing of ANC initiation, and previous history of ANC follow up were found to be associated with late initiation of ANC. However, in a multivariable logistic regression, maternal age, marital status, unplanned pregnancy, perceived timing of first ANC visit, and previous ANC follow up were found to be significantly associated with late initiation of ANC after adjusting for confounders.

The odds of late ANC initiation were 25% lower among women with the age of 30 and above compared to other age groups (AOR = 0.25, 95% CI 0.06, 0.83). The odds of late first ANC visit were 2.8 times higher among married women than single women (AOR = 2.81, 95% CI 1.29, 6.09).

Pregnant women with an unplanned pregnancy were 2.17 times more likely to start their ANC late compared to those women with planned pregnancies (AOR = 2.17, 95% CI 1.4, 4.67).

Pregnant women who perceived that the correct time of ANC booking was after 12 weeks of gestation were 8.58 times more likely to delay the ANC initiation compared to those who perceived that correct time of ANC booking was before 12 weeks of gestation (AOR = 8.58, 95% CI 5.75,14.80). Pregnant women who had no ANC for previous pregnancy were 5.45 times more likely to start their ANC lately compared to those who had ANC for previous pregnancy (AOR = 5.45, 95% CI 3.02, 9.83) (Table 5).

**Table 5:** Factors associated with late initiation of first ANC at selected health centers in Addis Ababa, Ethiopia 2021(n = 401)

Factor	Timing of ANC initiation		Odds ratio with 95% CI		P-value
	Late (%)	Early (%)	COR	AOR	
Age (in years)					
15–19	13 (6.9)	13 (6.1)	1	1	
20–24	34 (18.1)	60 (28.0)	1.83 (0.72, 4.24)	0.98 (0.29, 3.3)	0.97
25–29	46 (24.5)	72 (34.0)	1.61 (0.66, 3.67)	0.66 (0.20, 2.2)	0.50
30 and above	95 (50.5)	68 (32.0)	0.57 (0.31, 0.83)	0.25(0.06, 0.94) *	0.02
Marital status					
Single	42 (22.3)	22 (10.0)	1	1	
Married	146 (78.0)	190 (89.0)	2.53 (1.42, 4.33)	2.8 (1.3, 6.1) ^*^	0.01
Educational Status					
Illiterate	55 (29.3)	48 (23.0)	1	1	
Primary education	80 (42.6)	64 (30.0)	0.92 (0.63,1.54)	1.05 (0.53, 2.1)	0.87
Secondary and above	53 (28.2)	101(47.0)	2.20 (1.31,3.62)	1.8 (0.8, 3.8)	0.12
Occupation					
Self employed	106 (57.0)	94 (44.0)	1	1	
Government employee	27 (14.5)	51 (24.0)	2.10 (1.23, 3.70)	1.43 (0.62, 3.30)	0.40
House wife	47 (25.3)	60 (28.0)	1.43 (0.89, 2.30)	0.87 (0.46, 1.62)	0.65
Others^**c**^	6 (3.2)	8 (3.8)	1.50 (0.50, 4.51)	2.20 (0.43, 11.12)	0.34
Planned pregnancy					
No	155 (83.3)	184 (86.4)	1	1	
Yes	31 (16.7)	29 (13.6)	1.27 (1.23, 5.12)	2.17 (1.44, 4.67) *	0.01
Transportation cost					
No	25 (13.3)	45 (21.0)	1	1	
Yes	163 (87.0)	168 (79.0)	0.62 (0.34, 1.14)	0.73 (0.42, 1.50)	0.38
Perceived timing of the first ANC visit					
≤ 12 weeks	69 (38.8)	173(83.2)	1	1	
> 12 weeks	109 (61.2)	35 (16.8)	7.80 (7.12, 30.71)	8.58 (5.75,14.80) *	0.01
Previous ANC follow up					
No	100 (54.0)	48 (23.0)	1	1	
Yes	85 (45.9)	164(77.0)	4.01(2.60, 6.23)	5.45(3.02, 9.83) *	0.001

c Daily laborer and housemaid

*P-Value < 0.05; *ANC* Antenatal care, *COR* Crude odds ratio, *AOR* Adjusted Odds ratio

## Discussion

Antenatal Care (ANC) is crucial for reducing maternal morbidity and mortality through early detection and treatment of pregnancy-related complications. The first ANC visit should be initiated as early as the pregnancy was confirmed or within 12 weeks of gestation. In our study, 46.9% of respondents booked their first ANC visit lately. This finding is consistent with other studies conducted in Addis Ababa (42.0%) [25], Jimma 48% [16], and Cameroon (44%) [29].

This study finding is higher compared to a study done in Debre Markos (33.4%) [23]. This variation could be due to the classification of the outcome. Because the study in Debre Markos classified mothers as late for booking ANC if they came after 16 weeks of gestation. In our study, however, late ANC has been considered if a pregnant woman started ANC follow-up after 12 weeks of gestation.

Yet, the prevalence of late ANC initiation in our study is lower than studies conducted in Tanzania (70.4%) [30], Zambia (72%) [31], and studies conducted in other parts of Ethiopia such as Ilu Ababor (71.2%) [27] and Tigray (85%) [24]. This improvement might be attributed to the current national emphasis given to the focused ANC, and the massive work done to improve maternal health outcomes in the country. Further, the sociocultural differences among the study populations might also contribute to the variation observed between our findings and the findings reported in previous studies.

In this study, pregnant women aged 30 years and above had lower odds of a late first ANC visit than other age groups. This might be because early pregnancy awareness is increased with maternal age. This finding is supported by the results of studies conducted in Ghana and other sub-Saharan African countries [32, 33].

This study revealed that the odds of a late first ANC visit were 2.8 times higher among married women as compared to those with a single marital status. The possible reason could be married women may feel more confident of their previous ANC experience, and familial or partner support, and may perceive that starting ANC follow-up lately may not impact their health and the health of their fetus. This is also supported by other study findings [16, 34, 35].

Pregnant women with an unplanned pregnancy were 2.17 times more likely to start their ANC late compared to those women with planned pregnancies. This might be because those women with an unplanned pregnancy may lack a support from their partners or families and become less motivated to seek ANC early compared to women with planned pregnancies. This finding is consistent with other previous studies reports [16, 18, 23, 36].

This study showed that the likelihoods of late ANC initiation were 8.58 times higher among women who perceived that the correct time of ANC initiation is after 12 weeks of gestation as compared to those who perceived that the correct time of ANC initiation is before 12 weeks of gestation. This finding is in line with the findings reported in studies conducted in Debre-Markos [23], Ilu Ababor [27], Addis Ababa [25], and southern Ethiopia [37].

This study indicated that previous ANC service is a strong predictor of late ANC initiation. The odds of late ANC booking were 5.45 times higher among women who had no ANC for previous pregnancy than those who had ANC for previous pregnancy. This might be related to the counseling and health education received on the importance of early initiation of ANC in the previous pregnancy. This finding is backed by the findings reported in studies conducted in different parts of Ethiopia [23, 27, 37].

### Limitation of the study

This study considered only pregnant women attending ANC at the governmental health institutions. However, other pregnant women who visit private clinics, hospitals and NGOs which focus on maternity care were not included in this study. Furthermore, since the study was cross sectional, it was not possible to establish the temporal relationship of variables.

## Conclusion

Almost half of the pregnant women booked their first ANC visit lately. Maternal age, marital status, pregnancy intention, perceived timing of ANC initiation and previous ANC service were identified predictors of late ANC initiation. All stakeholders should work more on awareness creation and addressing misperception, aiming to promote early ANC initiation among women who perceived wrong timing of first ANC, married women, women with unplanned pregnancy, and those who have no ANC for their previous pregnancy.

## Supplementary Information

**Additional file 1**. Data collection tool/Questionaire.

## Data Availability

All related data are presented fully within the paper. Additional data are available from the corresponding author on reasonable request.

## Abbreviations

ANC: Antenatal care
AOR: Adjusted odds ratio
CI: Confidence interval
EDHS: Ethiopian demographic and health survey
ETB: Ethiopian birr
NGOs: Non-governmental organizations
WHO: World health organization
