# Supplementary material for: Late initiation of antenatal care among pregnant women in Addis Ababa city, Ethiopia: a facility based cross-sectional study
Source: BMC Womens Health. 2023 Jan 10;23:13. doi: 10.1186/s12905-022-02148-4 (PMC9832813; doi:10.1186/s12905-022-02148-4)
Supplement: Supplementary file 1 — Additional file 1. Data collection tool/Questionaire. [file 12905_2022_2148_MOESM1_ESM.docx]

**Consent form**

Dear participants, I am ----------------------------, data collector for the study entitled “late initiation of Antenatal care and associated factors among Antenatal care attendants in selected Health Centers of Addis Ababa, Ethiopia’’. Your participation in the study is important input for the completion of the study. There may not be direct benefits to you for giving us information for the study, but your participation will help us to provide information for planners and concerned stakeholders in order to improve maternal health care service and fill the gap associated with late ANC initiation. Participation in this study is voluntary. You have the right to with draw from the study at any time and this will not label you for any loss of basic services. The information, which you provide will be kept confidential and there will be no information that will identify you. The maximum of 25 minutes is required to complete the interview. You are free to ask for any clarification on questions that you do not understand at any point or during the interview. Hence I kindly request you to lend me few minutes from your time to respond for the interview questions.

Are you volunteer to participate in the study?

Yes

No

I heard the information on the consent sheet & understood the purpose and benefit of the study.

I witness that I agreed to participate in the study with my signature below.

Participant's signature­­­­­­­­­­­­­­­­­­­­­­­___________________________

Interviewer name: ________________________ signature__________

Supervisor Name: ___________________Signature____________

If you have any questions or enquiries at any time about the study, please contact and communicate Mr. Niguse Girma

Address- Addis Ababa, Ethiopia Cell phone: +251917187261

E-mail: [nigusegirma206@gmail.com](mailto:nigusegirma206@gmail.com)

**Questionnaire**

| **No** | **Questions** | **Response** | **Code** |
| --- | --- | --- | --- |
| **Socio demographic variables** | | | |
| 101 | Age in years | --------------------- |  |
| 102 | Ethnicity | 1.Amhara  2.Oromo  3. Gurage  4. Tigray  5. others (specify)……. |  |
| 104 | Marital status | 1.Single (never married)  2. married (living together currently) |  |
| 105 | Educational Level | 1.Illitrate  2.Primary (1-8)  3.Secondary (9-10) and above |  |
| 106 | Occupation | 1 self. Employee  2.Government Employee  3.Housewife  4.Others (specify)……………. |  |
| 107 | Average Income per month | 1.Less than 500 birr  2.501-1000 birr  3.1001-1500 birr  4.greater than 1500 birr |  |
| 108 | Transportation cost paid for getting health service | 1.No  2. Yes, (specify) --- ETB |  |
| **Obstetric history** | | | |
| 109 | Gravida | 1.Number of pregnancies-------------  2.Number of abortions----------------- |  |
| 110 | Parity (Number of births) | 1.Number of children alive ----------  2.Number of children died-----------  3.Number of stillbirths---------------- |  |
| **Perception towards ANC** | | |  |
| 111 | How do you rate the importance of ANC for your health? | 1. Highly important  2. Medium  3. Less  4. Do not know |  |
| 112 | How do you rate the importance of ANC for the fetus? | 1. Highly important  2. Medium  3. Less  4. Do not know |  |
| 113 | When do you think is appropriate time to begin the first ANC visit once the pregnancy is confirmed? | 1. Before or at 3 months (12 weeks) 2. After 3 months (12 weeks) |  |
| 114 | How many times do you think a woman needs to go for ANC in a health facility during pregnancy | 1. One Visit  2. Two to Three Visits  3. Four to Six Visits  4. More than Six Visits |  |
| **Past history of service utilization** | | |  |
| 115 | Have you ever attended ANC? | 1. Yes 2. No |  |
| 116 | If yes, for Q 16, for which pregnancy you attended? | 1^st^ pregnancy |  |
|  |  | 2^nd^ pregnancy |  |
|  |  | 3^rd^ pregnancy |  |
|  |  | 4^th^ and above 1. |  |

| **History of current pregnancy** | | |  |
| --- | --- | --- | --- |
| 117 | \| How do you know your pregnancy? \|  \| \|  \| \| --- \| --- \| --- \| --- \| \|  \| \|  \| \| \|  \| \|  \| \| | 1. Missed period once  2. Missed period twice  3. Missed period more than three |  |
|  |  | 4.Physiological changes |  |
|  |  | 5.Other signs like nausea |  |
|  |  | 6. By examination [urine test] |  |

| 118 | Is this pregnancy planned? | 1. Yes 2. No |  |
| --- | --- | --- | --- |
| 119 | If this pregnancy is planned, did the decision include your husband? | 1. Yes 2. No |  |
| 120 | To whom did you say that you became pregnant for the first time? | 1. Your Husband  2. Your Mother  3. Your Sister  4. Your Friend  5. Other (specify)----------------- |  |
| **History of current ANC** | | |  |
| 121 | Before your first attendance of ANC, was there any one who advised you on the time to start ANC follow up? | 1. Yes  2. No |  |
| 122 | If you are advised on the time to start ANC follow up, when does he/she advised you to start your first ANC visit? | _________________ months after amenorrhea |  |
| 123 | If you attended ANC, when did you started ANC Follow up for the recent pregnancy? | __________________ months after last menstruation. |  |
| 124 | Why you decided to start (begin) the follow up at this time? | 1. It is appropriate time  2. From previous Experience  3.presence of pregnancy danger sign  4. other reasons (specify)----------- |  |
